# Editorial: Roles of non-coding RNAs in tumor growth and development

**DOI:** 10.3389/fonc.2022.1098315

**Published:** 2022-12-01

**Authors:** Gunjan Sharma, Isaia Barbieri, Giovanni Blandino, Valeria Poli, Jawed Akhtar Siddiqui

**Affiliations:** ^1^ Department of Biochemistry and Molecular Biology, University of Nebraska Medical Center, Omaha, NE, United States; ^2^ Molecular Biotechnology Center, University of Torino, Torino, Italy; ^3^ Translational Oncology Research Unit, IRCCS Regina Elena National Cancer Institute, Rome, Italy; ^4^ Fred and Pamela Buffett Cancer Center, University of Nebraska Medical Center, Omaha, NE, United States

**Keywords:** non-coding RNAs, microRNAs, cancer, metastasis, enhancer RNAs (eRNAs)

## Introduction

In the past two decades, high-throughput sequencing techniques tremendously improved our understanding of the non-coding transcriptome, including the discovery, functions, and regulation of non-coding RNAs (ncRNAs) in normal and pathological conditions. Indeed, only 3% of RNAs are translated into proteins, whereas 75% of the human genome is transcribed. ncRNAs are an extremely diverse class of RNA molecules deeply involved in regulating gene expression, specialized cells functions, proliferation, and differentiation. As such, it is not surprising that their dysregulated expression or mutation can trigger pathological phenomena, including cancer, affecting many different biological processes. Therefore, a more detailed understanding of their regulation, functions, and underlying molecular mechanisms may help in developing novel disease markers and targeted therapies.

This editorial summarizes the recent overview, significant findings, and perspectives provided in 20 research, mini-review, and review articles published over the past few months in the Frontiers Research Topic *Role Of Non-coding RNAs In Tumor Growth And Development*.

MicroRNAs (miRNAs) and long non-coding RNAs (lncRNAs) are the most abundant and best-characterized classes of regulatory RNAs. Briefly, microRNAs are short double-stranded RNAs that down-regulate the expression of sets of target mRNAs, triggering translational repression but also indirectly inducing mRNA degradation. Their expression is driven by RNA Polymerase II as a hairpin-shaped precursor (pri-microRNA) that is processed *via* two consecutive maturation events occurring in the nucleus and in the cytoplasm. They are highly conserved among species and regulate sets of functionally correlated genes (1). On the other hand, long non-coding (lnc) RNAs, both intra-genic and inter-genic, are defined as nuclear-encoded RNAs longer than 200 bp that lack coding sequences, and they are poorly conserved across species (2). Despite their definition, it has been discovered that a number of lncRNAs contain short Open Reading Frames that are translated into functionally relevant peptides (3). Also, lncRNAs are transcribed by RNA Pol II as polyadenylated, intron-containing RNAs that undergo splicing. They can function both in cis and trans, in the nucleus or in the cytoplasm *via* many different mechanisms dictated by their locus, primary sequence and/or tridimensional structure (4).

Altered expression of both ncRNA classes often occurs in pathological conditions, including tumor onset and progression (5, 6). Here, we divided the contributions into review/minireview articles and research papers and subdivided them according to the class of ncRNA discussed.

## Reviews/minireviews

### LncRNAs

A number of review articles focus on various aspects of lncRNAs in cancer. In particular, Connerty et al. discuss the general role of lncRNAs in regulating cellular stress in cancer cells. The authors posit that exploiting lncRNAs-mediated regulatory mechanisms may be a quick and efficient way for cancer cells to overcome oxidative, metabolic, and genotoxic stress, one of the most complex challenges for cancer cells. Other authors discuss the roles of specific lncRNAs, such as DRAIRC (Downregulated RNA In Cancer, Yao et al., PCGEM1 (Prostate Gene Expression Marker 1, Su et al., and WT1-AS (Wilms tumor 1 antisense RNA, Zhang et al., in multiple cancer types. DRAIC (Yao et al.) is downregulated in many solid tumors, affecting cancer cell proliferation, autophagy, migration, and invasion. Its expression levels are highly associated with immune cell infiltration, tumor stage, lymph node metastasis, and overall survival of cancer patients, and the authors propose it as an attractive target for both diagnosis and treatment. In contrast, pro-oncogenic PCGEM1 (Su et al.) is consistently up regulated in clinical samples and tumor xenografts compared to normal tissue, affecting cancer cell’s motility and metabolism. Interestingly, this lncRNA is able to act both as a competing endogenous RNA (ceRNA) and as a scaffolding RNA to regulate cancer cell’s growth and apoptosis. Finally, Zhang et al. describe the role of the WT1-AS, a lncRNA antisense to the WT1 gene, in numerous malignancies and how its expression correlates with clinicopathological features such as tumor size, tumor-node-metastasis stage, and survival.

A peculiar sub-class of long non-coding RNAs is represented by circular RNAs, formed by back-splicing of coding mRNAs. To this end, Liu et al. discuss the regulatory network of circular RNA zinc finger RNA binding protein (CircRNA-ZFR) in cancer onset and progression, highlighting its aberrant expression in numerous solid cancers where it acts *via* multiple mechanisms.

### Lnc RNAs and microRNAs

Ghafouri-Fard et al. offer a comprehensive description of the many micro and lncRNAs whose expression is either up- or down-regulated in endometriosis, a disease condition where endometrial-like tissue grows outside the uterus that is known to represent a predisposing factor for the development of many types of cancer. These ncRNAs expression signatures have a strong potential as diagnostic tools in endometriosis, a relevant issue where specific markers are still lacking.

Both coding and non-coding RNAs have been detected in exosomes, where they are believed to play specific roles affecting the gene expression of target cells. Tumor exosomes display indeed highly specific exosome content. Chen et al. provide an overview of tumor-derived exosomal lncRNAs, highlighting their crucial role in mediating direct communication between cancer cells and immune cells, cancer-associated fibroblasts, and endothelial cells in the tumor microenvironment. In this vein, Qiu et al. elaborate on RNA sorting and packaging in secreted exosomes and their roles in cellular communication, discussing the different known mechanisms, biological functions, and potential clinical and therapeutic applications of exosomal microRNAs and lncRNAs.

### microRNAs

The oncological role, diagnostic/prognostic values, and molecular mechanisms of the emerging pro-oncogenic microRNAs -1269a and -1269b, essential members of the miRNA 1269 family, are discussed in multiple cancers the review by Xie et al.. The mini-review authored by Ma et al. focuses instead on the significance of miR-1224 in regulating tumor progression, metastasis, invasion, angiogenesis, and drug resistance in multiple solid cancers. Interestingly, while this miRNA is mainly believed to act as a tumor suppressor, it is sometimes involved in tumor progression through metabolic reprogramming of aerobic glycolysis.

## Research articles

Most research articles focused on the identification and characterization of different lncRNAs. For example, Congrains et al. characterized and validated a novel sense lncNR4A3 as a regulator of the tumor suppressor gene NR4A3, by modulating the RNA processing machinery components in myeloid malignancies. Zhang et al. report increased expression of lncRNA TMPO antisense RNA 1 (TMPO-AS1) in bladder cancer patient samples. They show that it acts by facilitating the interaction between the cell cycle regulatory transcription factor E2F1 and OUT domain-containing ubiquitin aldehyde binding 1 (OTUB1), triggering E2F1 deubiquitination and stabilization and promoting bladder cancer malignant features. Their rescue experiments established the essentiality of the TMPO-AS1/E2F1 axis in BC progression.

Enhancer RNAs (eRNAs), i.e., lncRNAs transcribed from enhancers’ genomic regions, are crucial in mediating transcriptional regulations usually of their cognate genes. From pancreatic adenocarcinoma (PAAD) transcriptomic data, Tong et al. identified the expression of the LINC00242 eRNA and its cognate gene PHF10 as positively correlating with better prognosis since lower expression correlated with tumor status and grade and low survival rate. LINC00242 and PHF10 expression levels also associated with the quality of the immune infiltrate and allowing to classify PAAD patients into high, medium, and low immune clusters.

The tumor-promoting role of circRNA SSU72 in thyroid malignancies was identified by Zhang et al. *via* CircBank Database bioinformatic analysis. The authors further validated its expression status in papillary thyroid carcinoma (PTC) tissue and cell lines and established the circSSU72/miR-451a/S1PR2 axis as crucial in PTC progression *via* functional *in vitro* experiments.

Despite the definition of lncRNAs as not encoding proteins, open reading frames (ORFs) and short-translated peptides were identified in several of them. Liu et al. developed a comprehensive database of lncRNA-encoded peptides called LncPep. This resource database provides a coding potential assessment for 883,804 lncRNAs across 39 species and represents a valuable tool for discovering and investigating novel functions of lncRNAs in cancer.

Li et al. explored Hox transcript antisense intergenic RNA (HOTAIR) lncRNA and its oncogenic functions in non-small cell lung cancer (NSCLC). They demonstrated that HOTAIR inhibits miR-149-5p-mediated suppression of NSCLC growth and proliferation, acting as an endogenous competing RNA for HNRNPA1 mRNA by sequestering miR-149-5p, thus promoting NSCLC progression.

Naipauer et al. analyzed the host and viral transcriptomes in cells and tumors infected with Kaposi’s Sarcoma-associated Herpes Virus (KSHV), to uncover the role of lncRNA-miRNA-mRNA driven networks in KSHV tumorigenesis. Their studies revealed differential expression of the cancer-related lncRNAs Malat1, Neat1, H19, Meg3, and their associated miRNA-target pairs, regulating various hallmarks of tumorigenesis such as cell cycle and p53 signaling.

BRCA1-Associated Protein 1 (BAP1) is known to enhance BRCA1 tumor suppressor potential and its inactivation is associated with various cancers, in particular clear-cell renal cell carcinoma (ccRCC). Liu et al. described a tumor-promoting role for the lncRNA NEAT1in BAP-1 deficient ccRCC, with NEAT1 acting as a ceRNA partner of SERPINE1 *via* shared miR-10a-5p.

In the last study included Moubarak et al. characterized miRNA-124a as a suppressor of brain and lung metastasis in melanoma, showing that its overexpression suppressed metastases formation without affecting primary melanoma growth *in vivo*.

Overall, the collection described here highlights the numerous and heterogeneous functions of ncRNAs in various cancers and their therapeutic potential. Nevertheless, many of the described mechanisms appear to be highly tumor type-specific and/or require further characterization at the molecular level. Further studies will help to draw a complete picture of the roles of ncRNAs in cancer, to be exploited in the clinics with novel diagnostic and therapeutic approaches. The next 10 years will certainly shed considerable light on the complex RNA expression networks contributing to determine many tumors biological and clinical features.

## Author contributions

GS and JS wrote the initial draft. All authors listed have revised and approved the final version for publication.

## Funding

This work and the authors are, in part, supported by grants from the U.S. Department of Defense (DOD) through the Prostate Cancer Research Program under Award No. W81XWH-21-1-0640 and Fred & Pamela Buffett Cancer Center (FPBCC) Support Grant (P30 CA036727) to JS, Italian Cancer Research Association (AIRC) IG 24851 to VP, AIRC Startup 26505 to IB.

## Conflict of interest

The authors declare that the research was conducted in the absence of any commercial or financial relationships that could be construed as a potential conflict of interest.

## Publisher’s note

All claims expressed in this article are solely those of the authors and do not necessarily represent those of their affiliated organizations, or those of the publisher, the editors and the reviewers. Any product that may be evaluated in this article, or claim that may be made by its manufacturer, is not guaranteed or endorsed by the publisher.
